# Numerical Simulation of In-Situ Direct Shear Test and Damage Failure Mechanism Study of Concrete-Bedrock Cementation Surface

**DOI:** 10.3390/ma18122718

**Published:** 2025-06-10

**Authors:** Hexin Ye, Jinlin Huang, Jianwei Zhang, Yu Lai, Kelei Cao, Yong Wang, Wenxuan Wang

**Affiliations:** 1 Guangdong Technology Center of Water Resources and Hydropower, Guangzhou 510635, China; yhexin2025@126.com; 2 Guangdong Research Institute of Water Resources and Hydropower, Guangzhou 510635, China; 3 School of Water Conservancy, North China University of Water Resources and Electric Power, Zhengzhou 450046, China; zhangjianwei@ncwu.edu.cn (J.Z.); z20231010053@stu.ncwu.edu.cn (Y.L.); caokelei456@163.com (K.C.); 13164367901@163.com (W.W.); 4 Pearl River Water Resources Research Institute, Pearl River Water Resources Commission, Guangzhou 510611, China; wycivil@126.com

**Keywords:** concrete gravity dam, concrete and bedrock cementation surface, in-situ direct shear test, failure mode, failure mechanism, numerical simulation

## Abstract

Owing to the insufficient understanding of the mechanical properties and damage mechanisms of concrete-rock bonding interfaces in dam foundations, this study establishes a refined three-dimensional simulation model for direct shear tests of concrete-rock bonding interfaces based on in-situ direct shear tests conducted at a reservoir. The damage evolution process and failure mechanisms of the concrete-rock interface under different loading conditions are investigated. The results indicate that under varying normal stresses, the shear stress-shear displacement curve exhibits an initial increase followed by a gradual decrease, with peak shear strength ranging from 1.074 MPa to 2.073 MPa and a maximum error of 8.48%, meeting engineering requirements. The damage evolution process of the concrete-rock interface under different normal forces was simulated and compared with in-situ direct shear test results, confirming the accuracy of the simulation. The failure modes of the concrete-rock interface under different loading conditions can be categorized into three types: bonding interface failure, mixed shear failure, and rock failure. The failure mode is closely related to the magnitude of normal stress—as normal stress increases, the area of shear fracture along the bonding interface expands, and the fracture surface becomes smoother. The findings provide a theoretical basis for the design, anti-sliding stability, and risk analysis of similar concrete gravity dams.

## 1. Introduction

The shear strength of the cementation surface of concrete and bedrock has an important impact on the safety of dams [1,2,3], especially under complex environmental conditions (cyclic loads, earthquakes, erosion, etc.). Cracks are easy to occur and gradually expand in the joint area of the dam structure; eventually, the penetration of cracks will lead to safety accidents such as dam instability [4]. The joint surface of concrete and bedrock is called the cementation surface, which is prone to sliding instability and failure due to its weak shear resistance. The accident will inevitably result in unpredictable losses, which should be paid enough attention to. Therefore, the research on the fracture damage of the cementation surface can not only help to reveal the instability mechanism of the hydraulic dam structure but also provide a theoretical reference for the operation safety and management of the dam, which has an important engineering significance [5,6,7].

Shear strength is an important mechanical index to characterize the fracture damage of the cementation surface of concrete and bedrock [8,9]. Most of the shear strength indicators are obtained through indoor or in-situ shear tests [10,11,12], among which in-situ shear tests can ensure the natural structural state of the rock material and reduce the disturbance during sampling and transportation, which can reflect the engineering reality more truly than indoor tests. Currently, many scholars at home and abroad have carried out a large number of in-situ direct shear tests for different geological conditions [13,14,15] and have achieved rich research results. Sanei et al. [16] conducted in-situ direct shear tests on rock discontinuities under constant normal load. They confirmed that the peak shear strength decreases with the increasing length of the discontinuity. Mouzannar et al. [17] showed that the peak shear strength of the cementation surface depends mainly on the morphology of the rock and the normal load. Ren et al. [18] conducted direct shear tests on rock samples to reveal the effect of discontinuous joints at different dip angles on rock shear damage. Zhang et al. [19] conducted in-situ shear creep tests, obtained the shear creep rate characteristics and its deformation law, which provide an important mechanical basis for the analysis and design of the dam base anti-slip stability. Guo et al. [20] found out the key factors affecting the results of shear tests and the damage characteristics by analyzing the results of in-situ shear tests on rock bodies with different degrees of weathering. In summary, it can be seen that the in-situ test reflects the actual characteristics of the rock mass and the real shear strength of the measured points; therefore, the test results are relatively realistic and reliable. However, considering the complexity and high cost of the equipment and the difficulty in observing the process of crack emergence, expansion, penetration, and destabilization inside the cementation surface [21,22], further investigation is still needed. With the rapid development of the computer field, numerical simulation technology has become an effective way to make up for the shortage of traditional experimental tests and theoretical analysis; meanwhile, it is also an effective means to study the mechanical properties and damage evolution process of cementation surfaces [23,24]. Reasonable simulation results can confirm, supplement, and explain the test to obtain more accurate conclusions.

Compared with in-situ tests, numerical simulation is convenient, fast, and low in cost and can simulate complex processes, which has been widely used in scientific research and engineering applications [25,26]. Lee et al. [27] established a plastic damage model of concrete structures under cyclic loading and obtained reliable simulation results. Geers et al. [28] used the damage model to simulate the damage and crack development of single-side notched beams and analyzed the influence of the tension-compression sensitivity of concrete on crack propagation. Dong et al. [29] conducted numerical simulations of crack extension in a series of concrete-rock combination beams and investigated the effects of roughness and natural cracking on the mechanical and fracture properties of the cementation surface, providing a theoretical basis for structural design and analysis. Tan [30] verified the effectiveness and rationality of the numerical model by comparing it with the results of the in-situ direct shear test and revealed the mechanical properties and progressive failure mechanism on the structural plane from a microscopic point of view. Yu et al. [31] reviewed the research progress of the concrete damage constitutive model at home and abroad and provided a reference for establishing a more perfect concrete damage constitutive model. Hu et al. [32] studied the mechanical properties of the contact surface between different materials and the applicability of the constitutive model through the numerical simulation of the progressive failure of the contact surface. Zhao et al. [33] pioneered an integrated approach combining digital image correlation (DIC) and acoustic emission (AE) techniques to investigate the interfacial bonding characteristics between engineered cementitious composites (ECC) and coal gangue concrete, establishing a novel methodological framework for composite interface analysis. Fan et al. [34] conducted systematic investigations on the shear transfer mechanisms at stabilized dredged soil-concrete interfaces, providing fundamental insights into soil-concrete interaction phenomena. Xie et al. [35] employed advanced PFC3D discrete element modeling to quantitatively analyze the meso-macro relationships in concrete direct shear tests, revealing significant scale effects in interface behavior. Liu et al. [36] performed comprehensive parametric studies examining surface roughness effects on the shear transfer capacity of magnesium potassium phosphate cement mortar-concrete interfaces. Yang et al. [37] developed a coupled experimental-numerical approach combining laboratory shear tests with discrete element simulations to characterize sandstone-concrete structural plane behavior. Sun et al. [38] established quantitative relationships between AE signature parameters and failure modes in sandstone joint shear tests, proposing a novel damage assessment methodology. Lu et al. [39] systematically evaluated the shear performance of interfaces between early-strength self-compacting shrinkage-compensating high-performance concrete (ESS-HPC) and existing concrete substrates. Wu et al. [40] conducted comparative studies on magnesium phosphate cement concrete-normal concrete interfaces, identifying key parameters controlling interfacial bond strength. Qin et al. [41] developed a refined discrete element framework for mesoscale parameter calibration in concrete shear simulations. Liu et al. [42] elucidated particle size-dependent roughness effects on sand-concrete interface shear behavior through controlled experimental programs. Wu et al. [43] characterized the bond-slip relationships of non-autoclaved ultra-high performance concrete (UHPC)-normal concrete interfaces under various loading conditions. Liu et al. [44] derived empirical formulations for predicting peak shear strength at rock-concrete interfaces based on extensive experimental databases. Zhao et al. [45] proposed a generalized Patton-type shear model incorporating surface morphology characteristics for rock-concrete joints. Zhang et al. [46] developed a thermo-hydro-mechanical coupled constitutive model for warm frozen soil-cast-in-place concrete interfaces. Li et al. [47] conducted comparative shear tests quantifying performance differences between silt-concrete interfaces and pure silt specimens. In summary, current research primarily focuses on slope rock masses or structural planes containing weak interlayers, mainly analyzing their experimental characteristics and stability. However, studies targeting the damage evolution, failure modes, and failure mechanisms of concrete-bedrock cementation surfaces remain relatively limited. While in-situ testing serves as a reliable method for obtaining the shear strength of cementation surfaces, its high costs and observational constraints make it difficult to fully reveal the failure mechanisms. Numerical simulation can effectively compensate for these experimental limitations by reconstructing crack propagation processes through damage models, thereby providing mechanical references for engineering design.

Based on in-situ direct shear tests of a reservoir, direct shear tests were conducted on the bonded interface between dam foundation concrete and bedrock under different normal loads (0.28 MPa, 0.58 MPa, 0.88 MPa, 1.20 MPa, and 1.50 MPa). The stress-displacement curves of the results were analyzed. However, the experimental results could only provide a macroscopic understanding of the mechanical properties, failing to reveal the shear evolution process under shear loading. Therefore, a refined three-dimensional numerical model was established to simulate the loading process by applying stepwise loads. The study investigated the distribution patterns of mechanical properties, the evolution of damage and failure processes, and the failure modes at the bonded interface. This approach elucidates the damage evolution mechanisms and failure behavior, providing theoretical references for similar engineering projects.

## 2. In-Situ Direct Shear Test Principle and Test Process

### 2.1. Testing Overview

**Figure 1:**
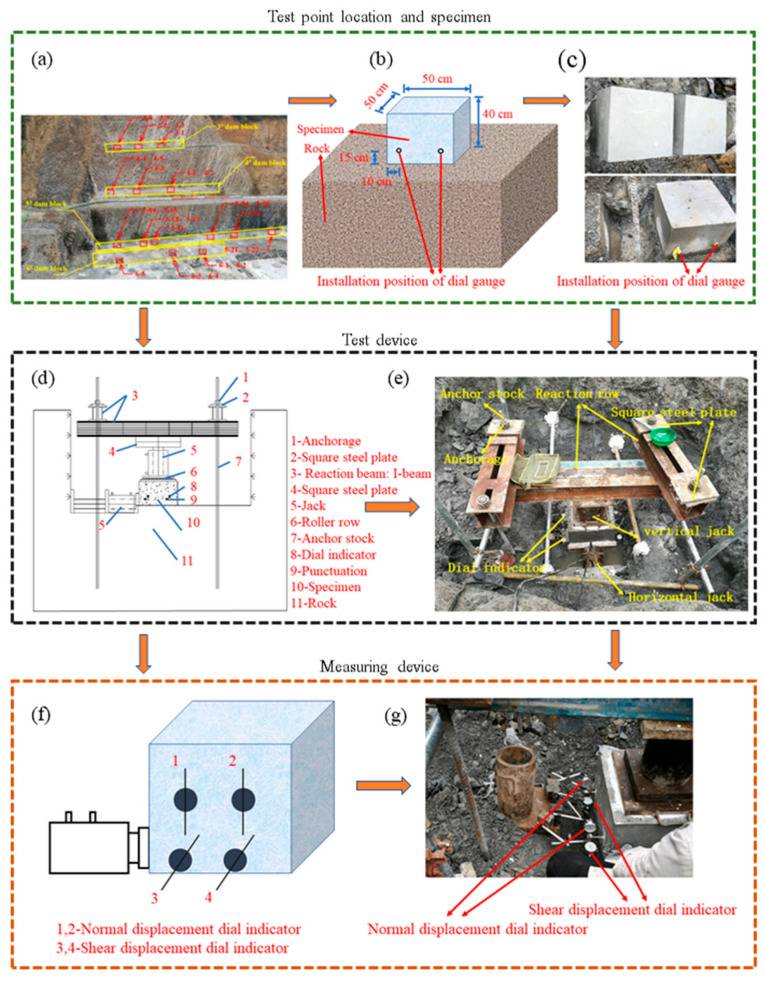
In-situ direct shear test device: (**a**) test point arrangement, (**b**) schematic diagram of specimen, (**c**) specimen preparation, (**d**) schematic diagram of loading device, (**e**) in-situ testing diagram, (**f**) sketch of installation of gauging system, (**g**) installation of gauging system.

The dam of Poyue Reservoir is a concrete gravity dam, with a height of 64 m, a crest length of 145 m, and a crest width of 6 m. The normal storage level of the reservoir is 385.5 m, and the total storage capacity is 1.73 million m^3^. The main task of the comprehensive water conservancy project is to supply water to downstream cities and towns for irrigation. To determine the mechanical parameters of the dam foundation rock mass, a direct shear test of the cementation surface between the concrete and the bedrock was carried out at the Poyue Reservoir dam site. The Poyue Reservoir dam is divided into 12 dam blocks from left to right, of which the non-overflow dam section on the left bank is divided into 5 dam blocks, from left to right are 1^#^~5^#^ dam blocks; the overflow dam section is divided into two dam blocks, 6^#^ and 7^#^ dam blocks from left to right; the non-overflow dam section on the right bank is divided into 5 dam blocks, from left to right are 8^#^~12^#^ dam blocks. In-situ direct shear tests were carried out on 3^#^, 4^#^, 5^#^, and 6^#^ dam blocks with a total of 5 groups (25 points); the test points and locations are shown in Table 1 and Figure 1a.

### 2.2. Testing Methodology and Procedures

The bottom surface of the specimens is square with a length of 50 cm and a height of 40 cm, as shown in Figure 1b,c. The experimental setup consisted of rock bolts, anchorages, square steel plates, and reaction beams as fixed components, along with separated hydraulic jacks for applying normal and shear loads and dial indicators for data measurement. The direct shear test is carried out until the concrete strength reaches the design strength of 20 MPa after natural curing for 28 days; the installation of the test equipment is shown in Figure 1d,e. Take the test on the 5^#^ dam block as an example; the normal loads (0.28 MPa, 0.58 MPa, 0.88 MPa, 1.20 MPa, and 1.50 MPa) are respectively applied to five specimens, and the normal load constant is then applied to the shear load step by step until the cementation surface reaches the shear failure standard [48]. The experimental procedure was conducted as follows: (1) Normal load application: The load was applied incrementally. Initial readings were taken immediately after applying the first load stage, followed by subsequent readings at 5 min intervals before proceeding to the next load stage. When the difference between two consecutive readings under the predetermined maximum load was ≤0.01 mm, indicating stabilized deformation, shear loading commenced. (2) Shear loading protocol: The applied shear load was divided into approximately 10 stages. Each load increment was maintained until stabilization before proceeding to the next stage, with comprehensive data recorded before and after each loading increment until the shear plane reached the failure criterion. (3) Post-test analysis: Upon completion of shear testing, the failure characteristics of the shear plane were documented and systematically analyzed. The loading and measuring device is shown in Figure 1f,g.

## 3. Numerical Simulation of the Direct Shear Test

### 3.1. Plastic Damage Constitutive Model of CDP Concrete

**Figure 2:**
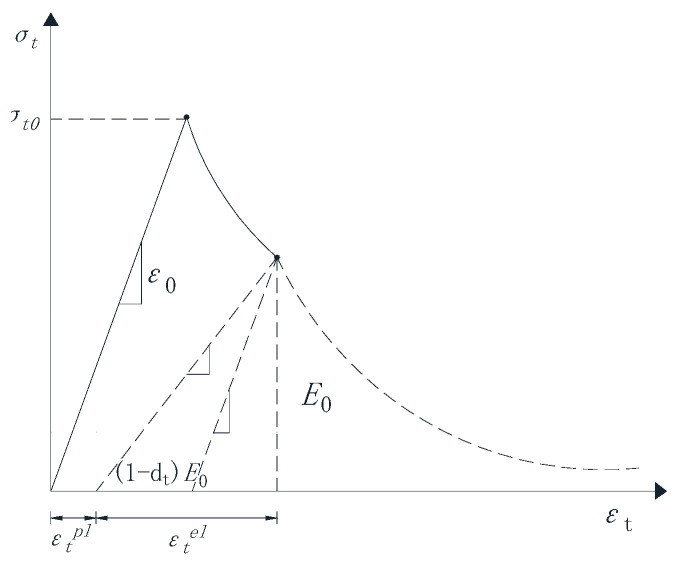
Stress-strain relationship curve of concrete under uniaxial tension.

**Figure 3:**
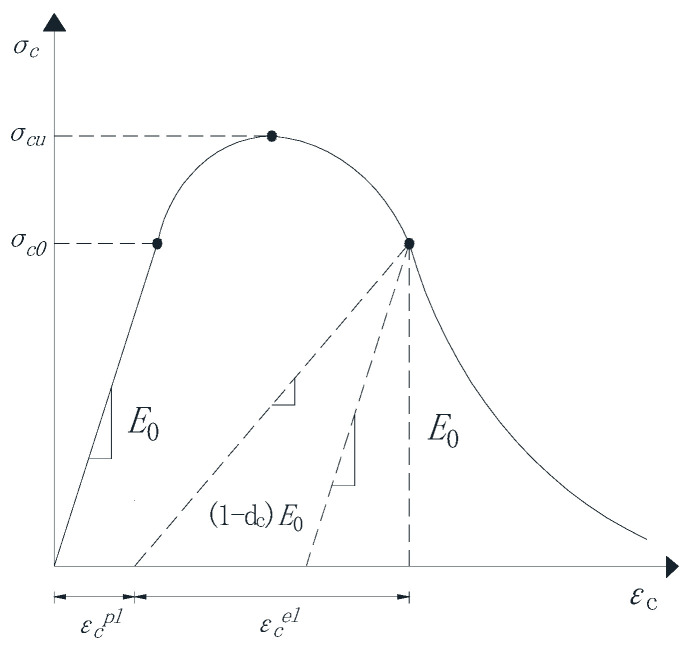
Stress-strain relationship curve of concrete under uniaxial compression.

The concrete plastic damage model used in this paper is based on the damage model proposed by Lubliner et al. [49] and improved by Lee and Fenves [27]. This damage model reflects the mechanical response and damage mechanism of concrete materials under cyclic loading or dynamic loading by introducing tensile and compressive damage factors and considering the difference in material tensile and compressive properties. The model assumes that concrete materials are mainly damaged by tensile cracking and compression fracture, which can be used for unidirectional, cyclic, and dynamic loading. This model is widely used to better describe the mechanical behavior of concrete under load. The uniaxial tension-compression stress-strain relationship of concrete is shown in Figure 2 and Figure 3.

The stress-strain relationship of concrete uniaxial tension and compression can be expressed by the following formula:

(1) $$\sigma_{t}=(1-d_{t})E_{0}(\varepsilon_{t}-\varepsilon_{t}^{pl})$$

(2) $$\sigma_{c}=(1-d_{c})E_{0}(\varepsilon_{c}-\varepsilon_{c}^{pl})$$

where $E_{0}$ is the initial elastic modulus; $d_{t}$ and $d_{c}$ are the tensile damage factor and compression damage factor, respectively; $\varepsilon_{t}^{pl}$ and $\varepsilon_{c}^{pl}$ are the elastic strains under tension and compression?

Under the action of external single or repeated loading, micro-cracks will form inside the concrete material, which will gradually expand and eventually be destroyed. At the same time, the material properties of concrete will gradually fail, which is called concrete damage. Based on the continuous damage mechanics theory (CDM), this process can be described by the damage variable D. The damage variable can be a tensor or a scalar, which mainly depends on the mechanical properties of the material being studied. Assuming that the mechanical properties of concrete materials are isotropic, then a scalar is used to represent the damage variable of the concrete, and its variation range is 0 ≤ D ≤ 1. When D = 0, it means that there is no damage to the concrete material; when D = 1, it means that the performance of the concrete material complete failure.

### 3.2. Mohr-Coulomb Failure Criterion

The bedrock is governed by the Coulomb failure criterion, wherein the shear stress (τ)–normal stress (σ) relationship curve is derived from the applied normal stress and its corresponding peak shear strength. Subsequently, the shear strength parameters are determined using Equation (1).

According to the Coulomb failure criterion [50], the formula for the peak shear stress of the direct shear test is

(3) τ=c+σtanφ

where τ is the shear stress, σ is the normal stress, c is the cohesive force, and φ is the friction angle.

### 3.3. The Simulation Model Establishment and Parameter Selection of the In-Situ Direct Shear Test

**Figure 4:**
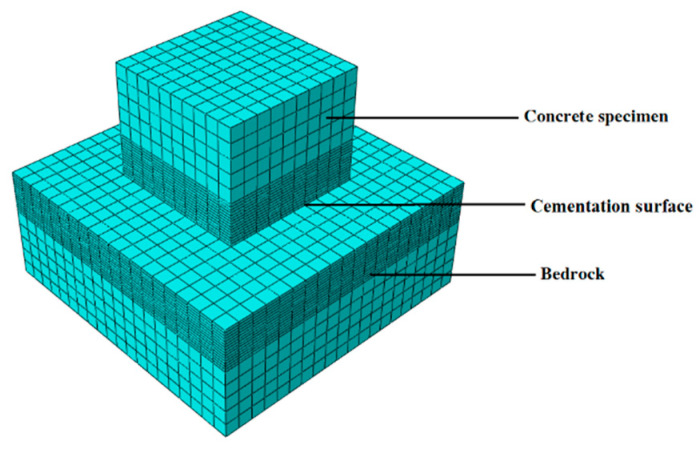
Numerical model of the in-situ direct shear test.

**Figure 5:**
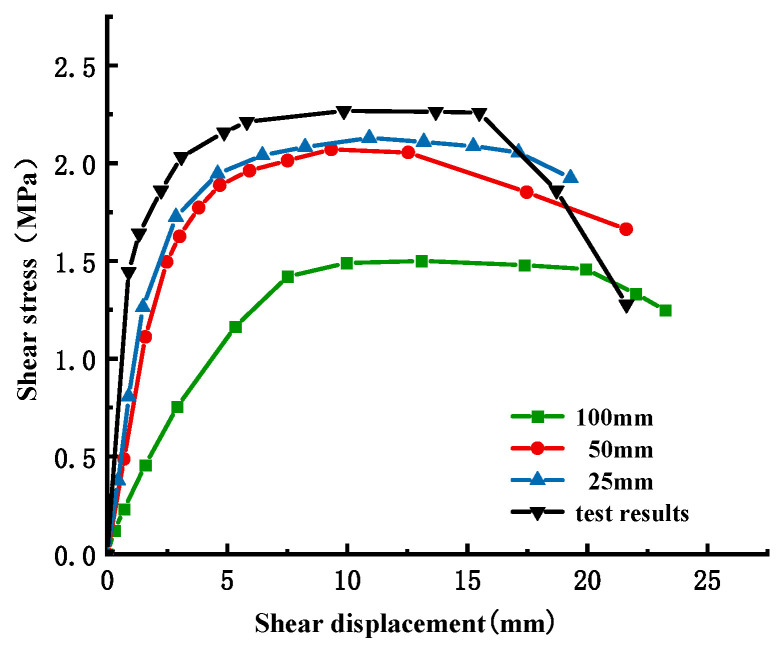
Comparison of simulated and experimental shear stresses under varying grid sizes.

**Figure 6:**
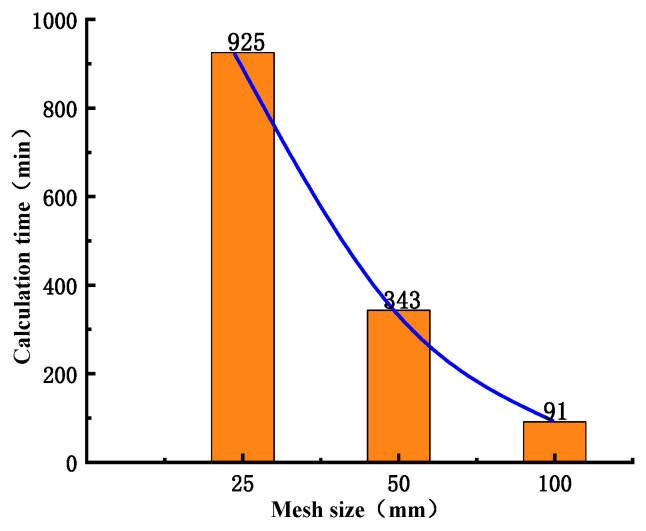
Calculation time under different mesh sizes.

Based on the engineering data and Figure 1, a three-dimensional refined in-situ direct shear test numerical simulation model is established (Figure 4), which reproduces the damage evolution process of the direct shear test and lays the foundation for revealing its damage mechanism. The concrete specimen size is 50 cm long × 50 cm wide × 40 cm high, and the bedrock size is 100 cm long × 100 cm wide × 40 cm high, and the material parameters are shown in Table 2. Additionally, normal constraints were imposed on the peripheral and basal boundaries of the bedrock section. To reduce the stress concentration phenomenon during the numerical simulation and improve the simulation accuracy, the model mesh adopts regular hexahedral cells, and the sensitivity analysis of the mesh size is carried out at the same time. The optimal mesh size is determined based on the simulation results (Figure 5 and Figure 6). It can be found that the peak shear stresses under the two sizes are 2.13 MPa and 2.07 MPa, and the errors from the test results are 8.4% and 6.1%, respectively, which all meet the calculation requirements, but the time varies greatly; it is recommended to choose a calculation model with a mesh size of 50 mm. The influence of mesh size on computational accuracy primarily stems from the localization of damage and plastic deformation within narrow zones (e.g., fracture bands or shear bands) when concrete undergoes tensile or compressive failure. Coarse meshes cannot adequately resolve localized high-strain gradients, resulting in smeared damage representation and artificial stress concentrations. This limitation becomes particularly pronounced in bending or shear failure modes, where standard elements fail to properly capture strain gradient effects. Combined with the test results, the mesh within 15 cm above and below the contact surface between the concrete and the bedrock is locally densified to further improve the simulation accuracy of the failure mode, and the refined mesh size is 10 mm. In the simulation calculation, multiple analysis steps are mainly set up to reproduce the loading process of the concrete-bedrock in-situ direct shear test, and the cumulative loading effect and damage evolution law of the specimen under load conditions are accurately characterized, which is helpful to reveal the damage evolution mechanism. The analysis steps of shear load under different working conditions are set as shown in Table 3.

## 4. Numerical Simulation Results and Experimental Verification

**Figure 7:**
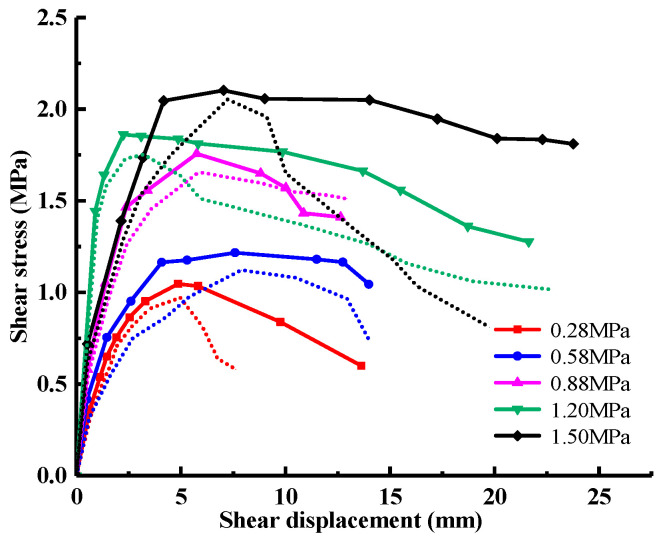
Shear stress-shear displacement curves under different normal stresses.

The shear stress-shear displacement relationship curve under different normal stresses obtained by numerical simulation is shown in Figure 7 (the solid line is the numerical simulation result, and the dotted line is the test result.). It can be seen from the figure that before reaching the peak shear stress, the slope of the curve decreases gradually with the increase in shear displacement; that is, the shear stress increases nonlinearly with the change of shear displacement. The reason for this phenomenon is that the local damage caused by the combined action of shear stress and normal stress reduces the overall shear strength of the cementation surface. The shear stress on the cementation surface decreases to a certain extent with the accumulation of damage, which indicates that the cementation surface is destroyed. Since then, due to the normal stress and friction on the cementation surface, the shear stress remains at a stable level. The error between the simulation results and the test results of peak shear strength under different normal stress conditions is 7.81%, 8.48%, 6.03%, 6.21%, and 2.44%, respectively, all of which are less than 10%, which meets the engineering requirements.

**Figure 8:**
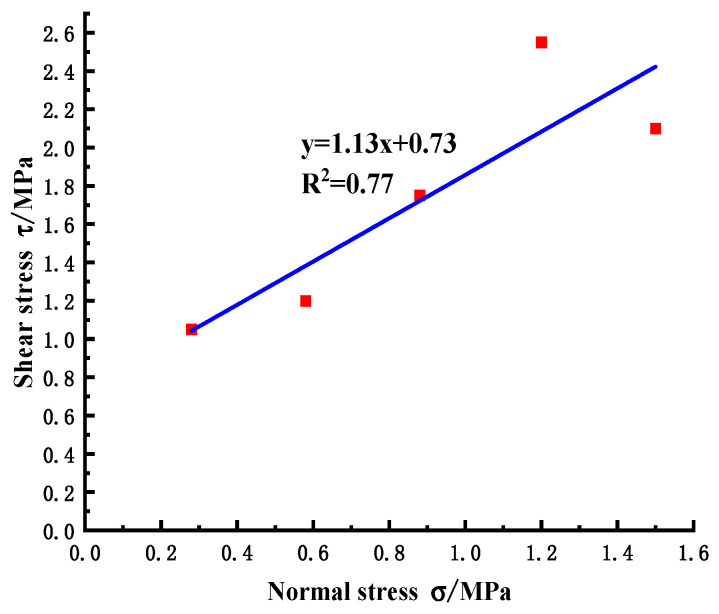
Normal stress-shear stress relationship diagram of numerical simulation.

**Figure 9:**
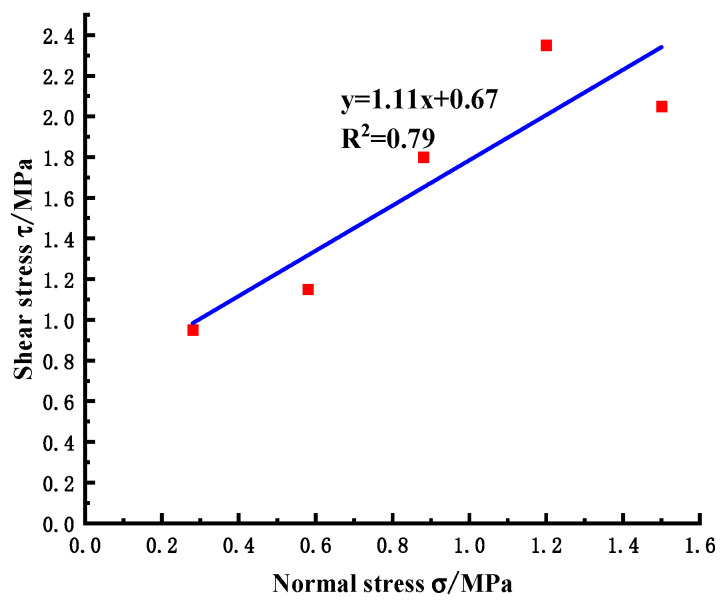
Normal stress-shear stress relationship diagram of the in-situ test.

By linear fitting of the peak shear strength under each normal stress, the slope and intercept of the line, namely the friction coefficient and cohesion, are obtained. It can be seen from Figure 8 that the friction coefficient is f = 1.13 and the cohesion is C = 0.73 MPa, which are in good agreement with the test results (Figure 9, the friction coefficient is f = 1.11 and the cohesion is C = 0.67 MPa). The errors are 1.77% and 8.22%, respectively, which meet the accuracy requirements and verify the effectiveness and accuracy of the simulation method.

**Figure 10:**
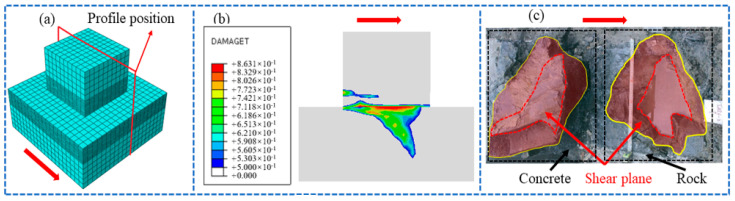
Fracture damage diagram of dam foundation concrete: (**a**) schematic diagram of profile position, (**b**) numerical simulation result, (**c**) test result.

To further verify the reliability of the simulation method, the damage cloud diagram of the cementation surface when the normal stress is 0.28 MPa is obtained by numerical simulation, as shown in Figure 10 (the arrow in the figure points to the shear direction, that is, the direction of shear load application, the same below). Compared with the cross-sectional view after the in-situ test, it can be seen that the development trend of the fracture surface is similar to the test result: the shear surface is mainly sheared along the cementation surface at both ends, which is relatively flat, and part of the rock mass is brought up in the middle, and the maximum fluctuation difference is about 10 cm. This result further verifies the effectiveness of the numerical simulation.

The slight discrepancy between numerical simulations and experimental results primarily arises from two fundamental factors: (1) the idealized material representation in simulations assumes concrete as a homogeneous, isotropic medium, whereas actual concrete exhibits inherent heterogeneity due to aggregates, voids, and interfacial transition zones; (2) the damage evolution in numerical models is governed by simplified mathematical formulations (e.g., exponential softening, linear softening), while real concrete damage involves more complex micromechanical behaviors influenced by stochastic microcrack propagation.

## 5. Analysis of Simulation Results of Direct Shear Test

**Figure 11:**
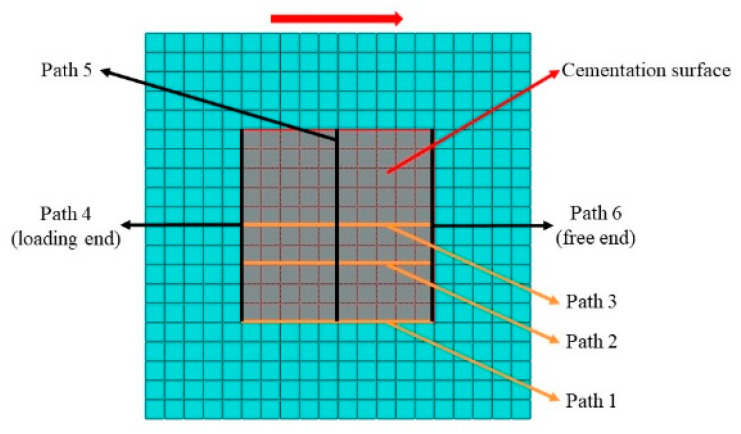
Path setting of cementation surface.

To facilitate the extraction of numerical simulation results, six paths at the edge of the cementation surface are selected, and the left end of the cementation surface is named the loading end, and the right end is named the free end, as shown in Figure 11 (top view).

### 5.1. Shear Stress Distribution on Cementation Surface

**Figure 12:**
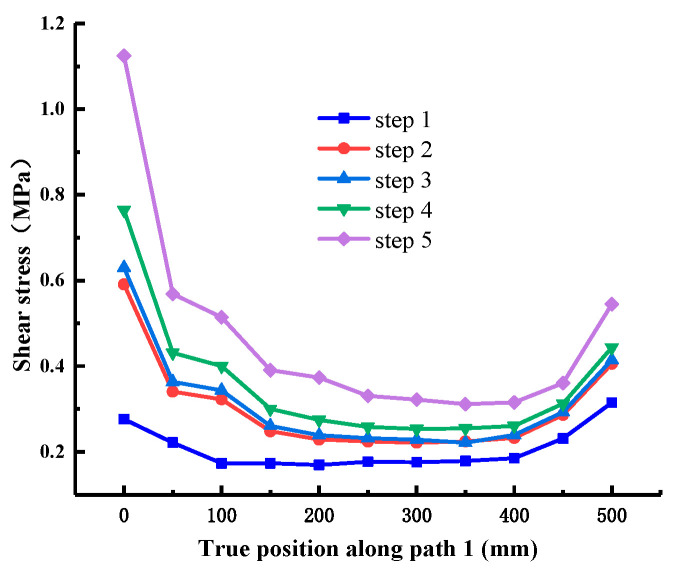
Shear stress at Path 1 (cementation surface).

**Figure 13:**
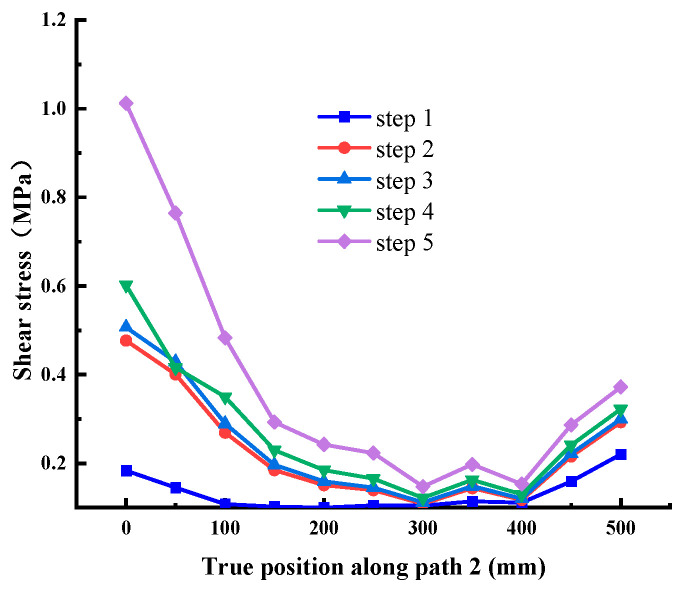
Shear stress at Path 2 (cementation surface).

**Figure 14:**
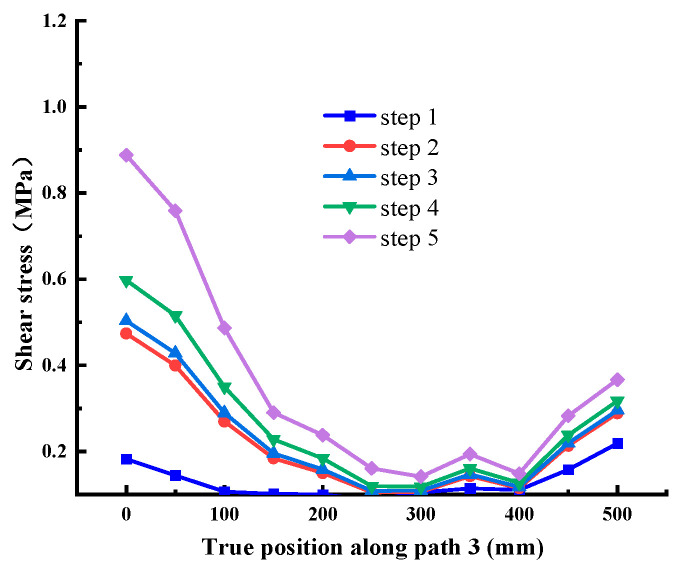
Shear stress at Path 3 (cementation surface).

**Figure 15:**
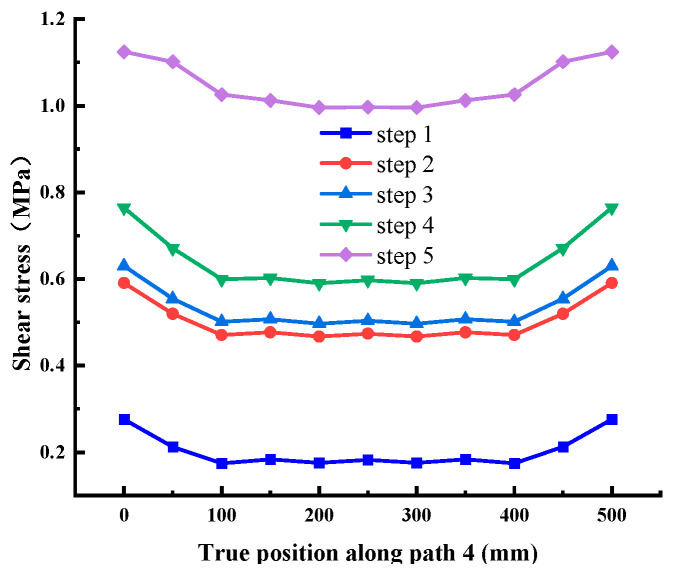
Shear stress at Path 4 (cementation surface).

**Figure 16:**
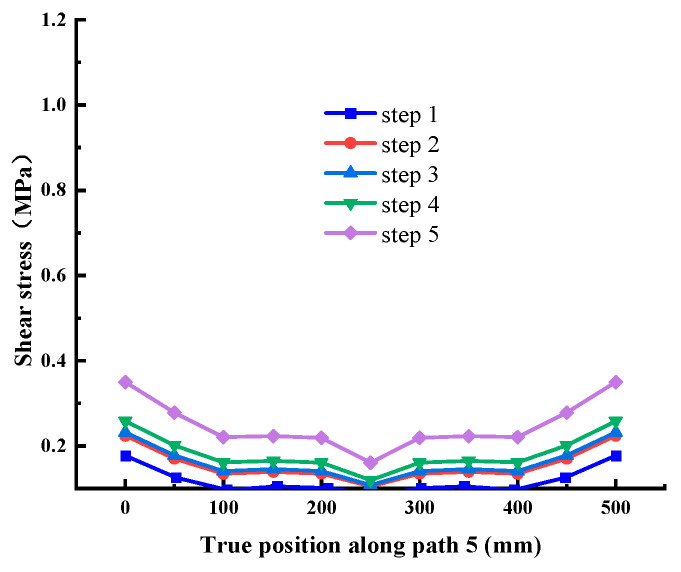
Shear stress at Path 5 (cementation surface).

**Figure 17:**
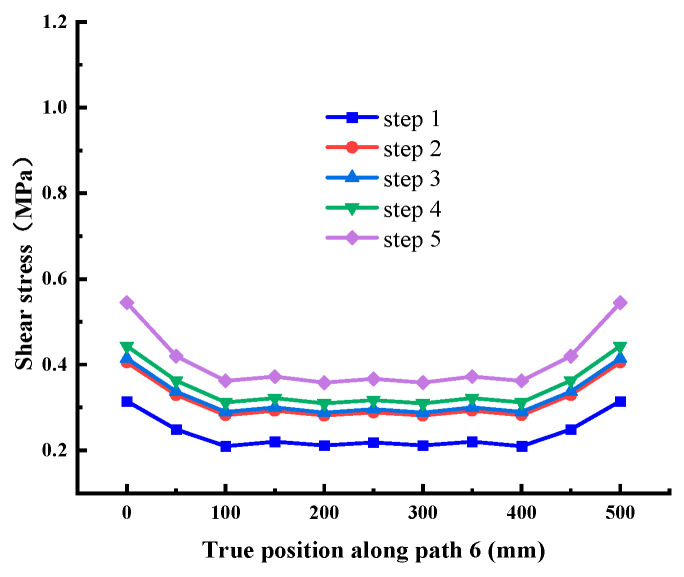
Shear stress at Path 6 (cementation surface).

When the normal stress is 0.28 MPa, the shear stress value is extracted along each path, as shown in Figure 12, Figure 13, Figure 14, Figure 15, Figure 16 and Figure 17. It can be seen from Figure 12, Figure 13 and Figure 14 that the stress distribution law of each path along the shear direction is consistent as a whole, with large ends and a small middle. With the increase in shear load, the concentration degree of shear stress becomes higher. The stress transfers from the loading end to the free end along the path, and the value gradually decreases. It tends to be stable in the middle part and increases in a small range before reaching the free end, indicating that stress concentration occurs at the loading end and the free end. At the early stage of loading, the specimen is in the elastic stage, the stress increases obviously with the shear load, and there are abrupt changes at the loading end and free end of each path; at the middle of loading, it shows the trend that the load increases but the shear stress increases less obviously, which indicates that it is beyond the elastic range of the material and enters the elastic-plastic stage; at the end of loading, the cementation surface has strong shear resistance, which makes the shear stress value suddenly change, reaches the shear failure standard, and the cementation surface is damaged. In addition, it can be seen from the figure that under the same load step, the shear stress value gradually decreases from path 1 to path 3, indicating that the edge area is the weak side of the cementation surface, and the cracks first occur at the low-strength point in this area.

It can be seen from Figure 15, Figure 16 and Figure 17 that the stress distribution of each path is relatively uniform in the direction perpendicular to the shear direction, and the stress at both ends is slightly larger than that in the middle area. With the increase in shear load, the shear stress at different positions on each path increases uniformly, which conforms to the general law. There is no abrupt change in stress transmission along this direction, and the shear stress of Path 4 and Path 6 is significantly greater than Path 5. Combined with the above conclusions, it shows that the stress distribution on the cementation surface is large at the edge and small in the middle.

**Figure 18:**
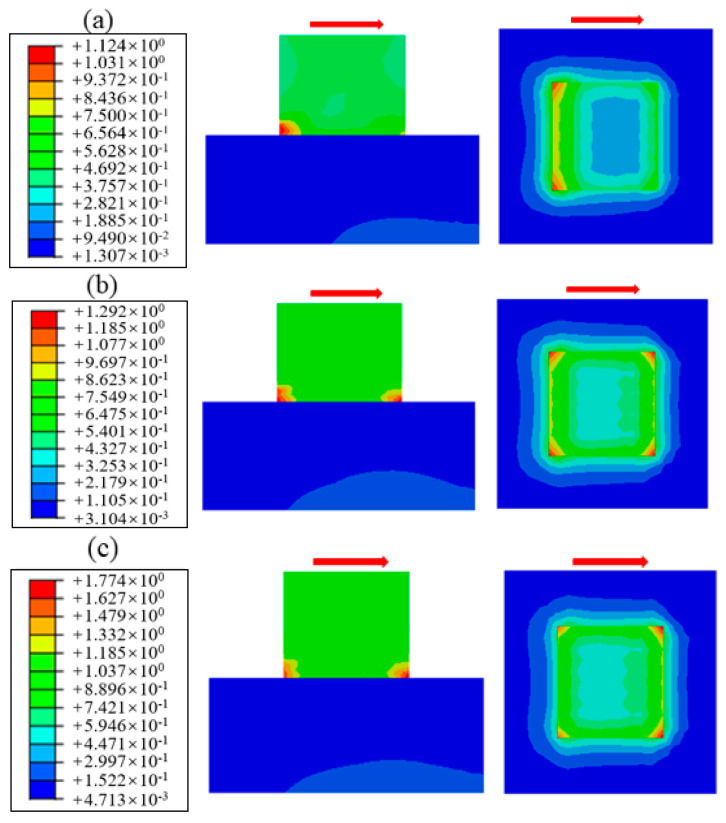

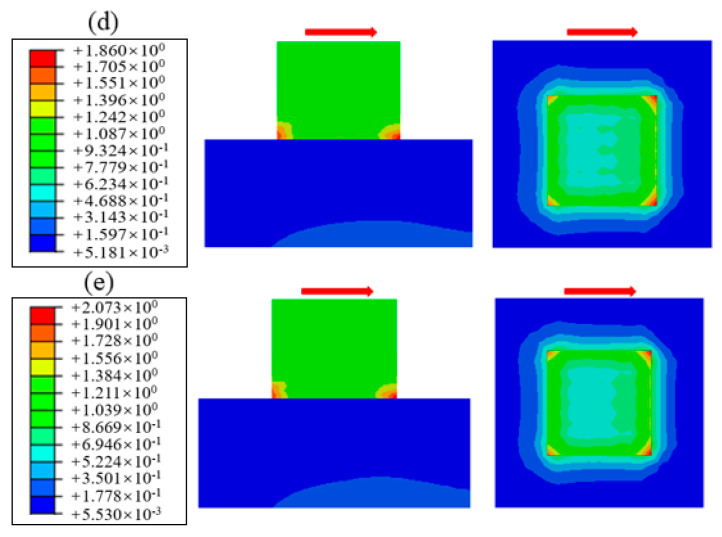
Shear stress distribution under different normal stress: (**a**) normal stress 0.28 MPa, (**b**) normal stress 0.58 MPa, (**c**) normal stress 0.88 MPa, (**d**) normal stress 1.20 MPa, (**e**) normal stress 1.58 MPa.

**Figure 19:**
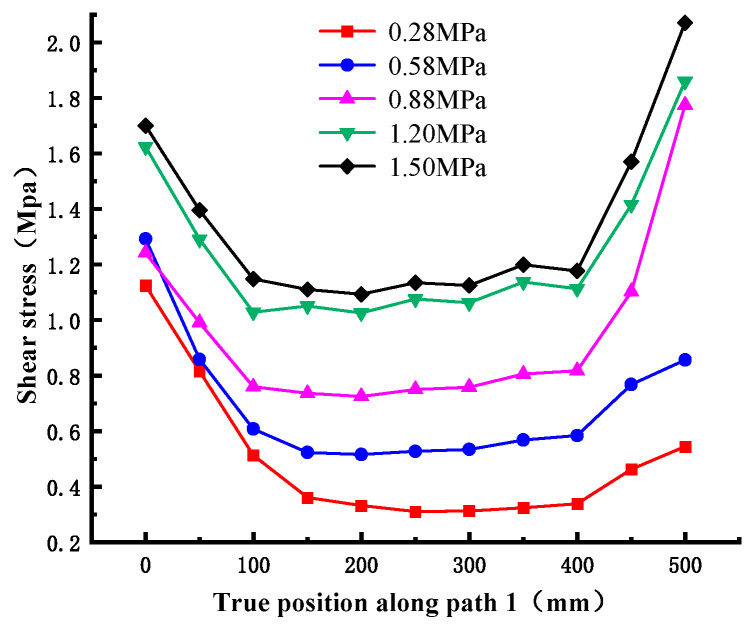
Shear stress value along path 1 under different normal stresses.

The cloud diagram of shear stress distribution is obtained by numerical simulation, as shown in Figure 18. By comparison, it can be found that the shear stress distribution under different normal stresses is generally consistent; the amplitude and range of the shear stress increase with the normal stress. In addition, it can be seen from the figure that the shear stress is unevenly distributed, which is larger at both ends and smaller in the middle, and the maximum shear stress is concentrated at the corners of the cementation surface. The underlying mechanism for this phenomenon stems from the absence of adjacent material constraints at free edges, which prevents uniform shear deformation transfer comparable to interior regions, consequently inducing localized shear stress concentration. The shear stress value under different normal stresses along path 1 is extracted, as shown in Figure 19. It can be found that when the normal stress is small, for example, the maximum shear stress corresponding to 0.28 MPa is 1.124 MPa, which appears on the loading end; when the normal stress increases to about 0.88 MPa, the maximum shear stress region begins to develop to the free end; for example, the maximum shear stress corresponding to 1.50 MPa is 2.073 MPa. It can be seen that when the normal stress is small, the shear damage mainly occurs near the loading end and extends to the free end until the specimen is completely fractured.

Through numerical simulation, it can be found that the stress distribution of the cementation surface is not uniform during the direct shear test. With the development of relative displacement, the element on the loading end is tensioned and generally fails first under the action of shear stress; the element on the free end is compressed with relatively high shear strength. In addition, stress concentration is prone to occur at the corners of the cementation surface, indicating that the damage may first occur at the corners during the test and then gradually expand to the middle part to form the through area. The reason for this phenomenon is caused by the existence of joints and cracks. The discontinuity and anisotropy of the rock mass are caused by the joints, which will change the mechanical properties of the rock mass under the different normal stresses; however, this mechanism is generally difficult to discover during the field test. Therefore, the stress distribution inside the specimen can be obtained by numerical simulation, the shear stress state and shear strength of the cementation surface can be predicted, and the mechanical properties of the cementation surface can be analyzed intuitively, which provides the theoretical basis for study.

### 5.2. Failure Analysis of Cementation Surface

#### 5.2.1. Failure Criterion

The failure criterion of the cementation surface in the numerical simulation mainly includes the following: the yield zone penetration method, the convergence criterion, the displacement mutation method, and the energy method. The loading process of the in-situ direct shear test is to apply the load step by step and record the shear displacement as the criterion of specimen failure, which is similar to the overload test of arch dams. Therefore, this paper refers to the criterion of the arch dam failure, that is, the yield zone penetration method is used to judge whether the cementation surface is damaged.

#### 5.2.2. Analysis of Failure Process

**Figure 20:**
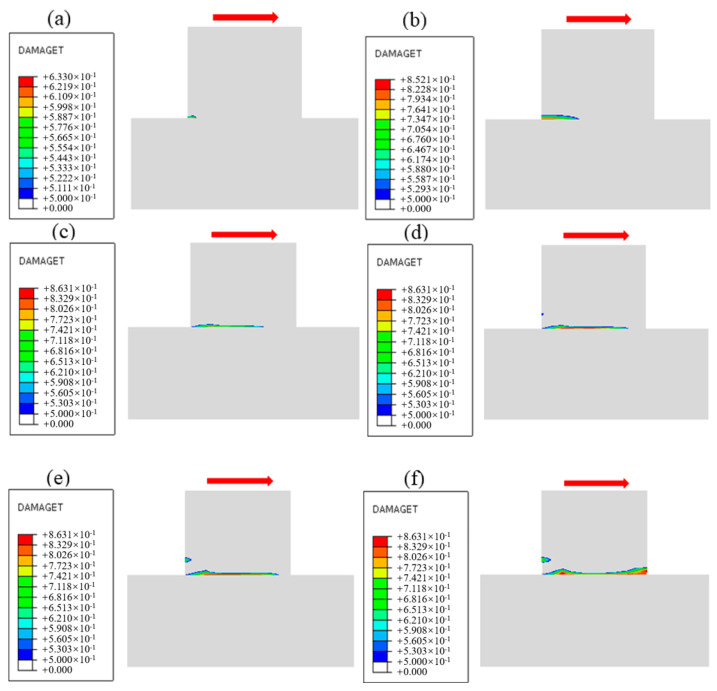
Damage evolution process when the normal stress is 1.50 MPa: (**a**) Time = 0.1 s, (**b**) Time = 0.3 s, (**c**) Time = 0.5 s, (**d**) Time = 0.7 s, (**e**) Time = 0.9 s, (**f**) Time = 1.0 s.

**Figure 21:**
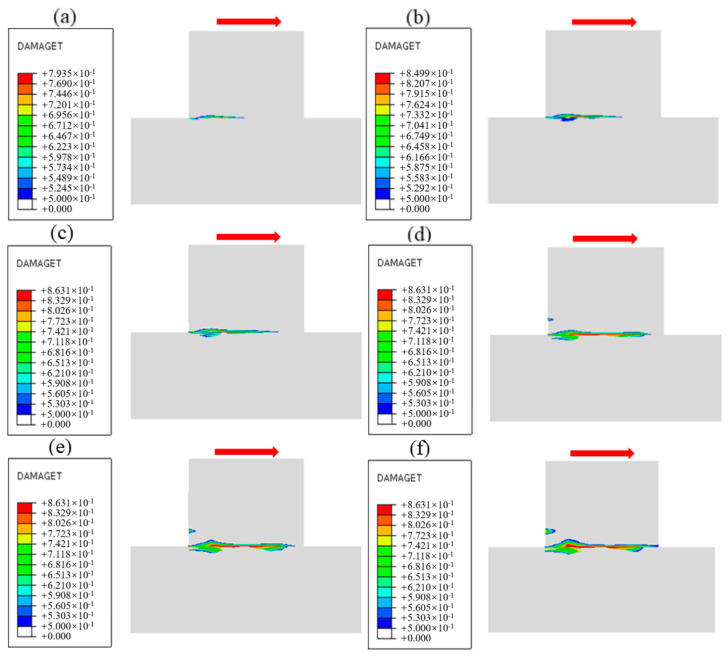
Damage evolution process when the normal stress is 0.88 MPa: (**a**) Time = 0.1 s, (**b**) Time = 0.3 s, (**c**) Time = 0.5 s, (**d**) Time = 0.7 s, (**e**) Time = 0.9 s, (**f**) Time = 1.0 s.

**Figure 22:**
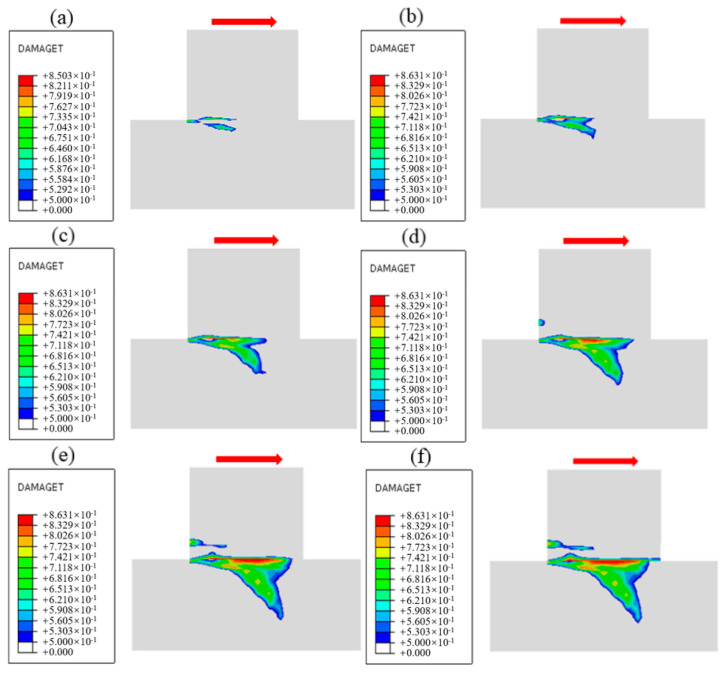
Damage evolution process when the normal stress is 0.28 MPa: (**a**) Time = 0.1 s, (**b**) Time = 0.3 s, (**c**) Time = 0.5 s, (**d**) Time = 0.7 s, (**e**) Time = 0.9 s, (**f**) Time = 1.0 s.

The damage evolution process near the cementation surface under different normal stress conditions is obtained by numerical simulation, as shown in Figure 20, Figure 21 and Figure 22, and the shear direction is indicated by the arrow. According to the literature [51], when the damage value is greater than 0.8, it is considered that the specimen will produce macroscopic cracks. To facilitate the observation of damage, the minimum damage value in the cloud image is set to 0.5. It can be seen from Figure 20 that the damage mainly develops along the cementation surface and first occurs at the loading end. With the increase in shear stress, the damage gradually extends from the loading end to the middle part until it passes through the entire cementation surface, indicating that the cementation surface of concrete and bedrock is completely damaged. It can be seen from Figure 21 that the damage first develops along the cementation surface. With the increase in shear stress, the damage begins to expand from the cementation to the lower bedrock, causing damage to the surface rock mass, whose failure surface is composed of the cementation surface and the bedrock. It can be seen from Figure 22 that the damage is mainly distributed in the bedrock, and the depth is large. With the increase in shear stress, the damage forms a through zone in the bedrock; that is, the shear failure mainly occurs in the bedrock. Combining with the results shown in Figure 7, it can be found that the reason for the nonlinear relationship between shear stress and shear displacement is due to the damage on the cementation surface, which also proves that the mechanical properties and failure mechanism of the cementation surface can be essentially revealed from the perspective of the damage evolution law.

**Figure 23:**
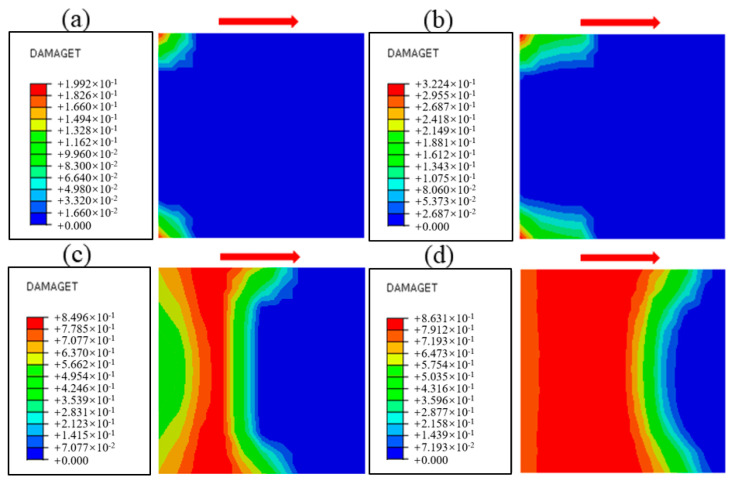

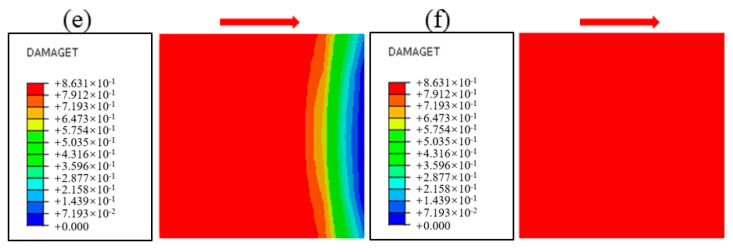
Damage evolution process of cementation surface when normal stress is 1.50 MPa: (**a**) Time 0.1 s, (**b**) Time 0.3 s, (**c**) Time 0.5 s, (**d**) Time 0.7 s, (**e**) Time 0.9 s, (**f**) Time 1.0 s.

**Figure 24:**
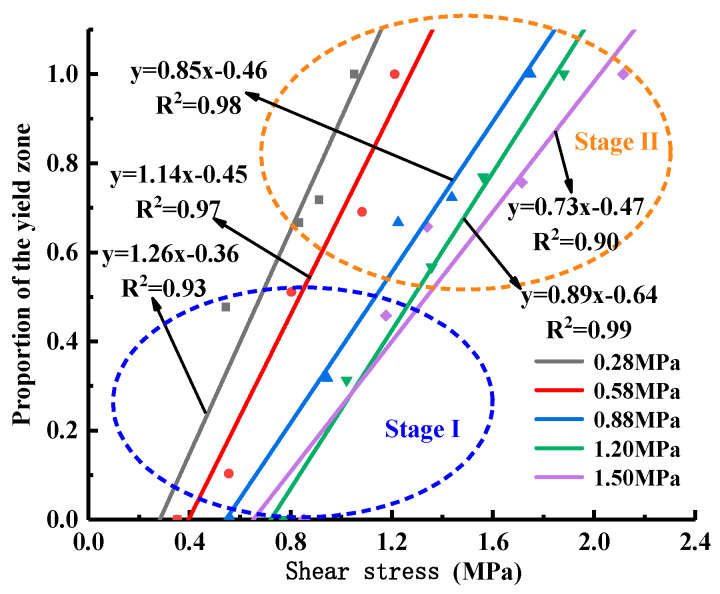
Proportional development curve of cementation surface yield zone under different normal stresses.

Figure 23 shows the damage cloud at the cementation surface when the normal stress is 1.50 MPa, where the red area is the yield zone, indicating that failure occurs there, and the blue area is the unyielding zone. The process by which the proportion of the yield zone of the cementation surface changes with the increase in shear stress under different normal stresses is shown in Figure 24, from which it can be seen that in the early stage of loading, Before the yield zone ratio reaches 0.6, that is, in stage I, the development trend of the yield zone at the cementation surface under different normal stresses is similar, the shear stress when the cementation surface begins to fail gradually increases with the increase in normal stress, the development of the yield zone is faster, the degree of damage in the cementation surface area under various working conditions increases uniformly, and gradually expands along the shear direction, when the yield ratio reaches 0.6, that is, in stage II, the development of the yield zone slows down, and with the increase in normal stress, the damage evolution rate of the cementation surface gradually decreases, and the maximum shear stress when the cementation surface is completely destroyed also gradually increases, indicating that the damage evolution rate of the cementation surface is related to the normal stress. To further understand the development trend of yield zone proportion, the data in the figure are linearly fitted. It can be seen from Table 4 that with the increase in normal stress, the slope of the straight line shows a decreasing trend, indicating that the shear strength increases with the rise of normal stress, and the correlation coefficients are 0.93, 0.96, 0.98, 0.99, and 0.90, respectively, indicating that there is a good correlation between the parameters.

The relationship curve obtained by regression analysis is calculated by the following equation, which can provide a reference for engineering to predict the damage development rate under different normal stresses and the corresponding shear stress when damage occurs:

(4) P=a×τ+b

where P is the yield zone ratio; τ is the shear stress, the fitting parameter a, b is related to the normal stress?

The relationship between fitting parameters and normal stress is shown in Equations (5) and (6).

(5) a=−0.43×σ+1.35

(6) b=−0.36×σ−0.13

However, during the in-situ direct shear test, it is difficult to obtain the damage to the cementation surface in the entire loading process; the relative displacement is generally used to judge whether the damage occurs. In addition, the damage to the shear plane can only be described and analyzed after the test. Through numerical simulation, the damage and destruction of each area and each measuring point can be analyzed, which makes up for the deficiency that the test can only collect the information of characteristic points and supplements the test results.

### 5.3. Analysis of Failure Mode and Failure Mechanism

**Figure 25:**
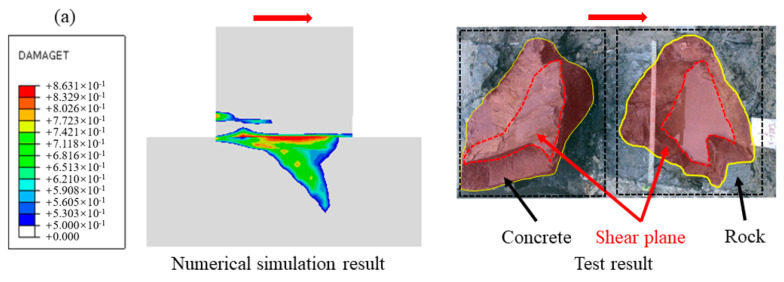

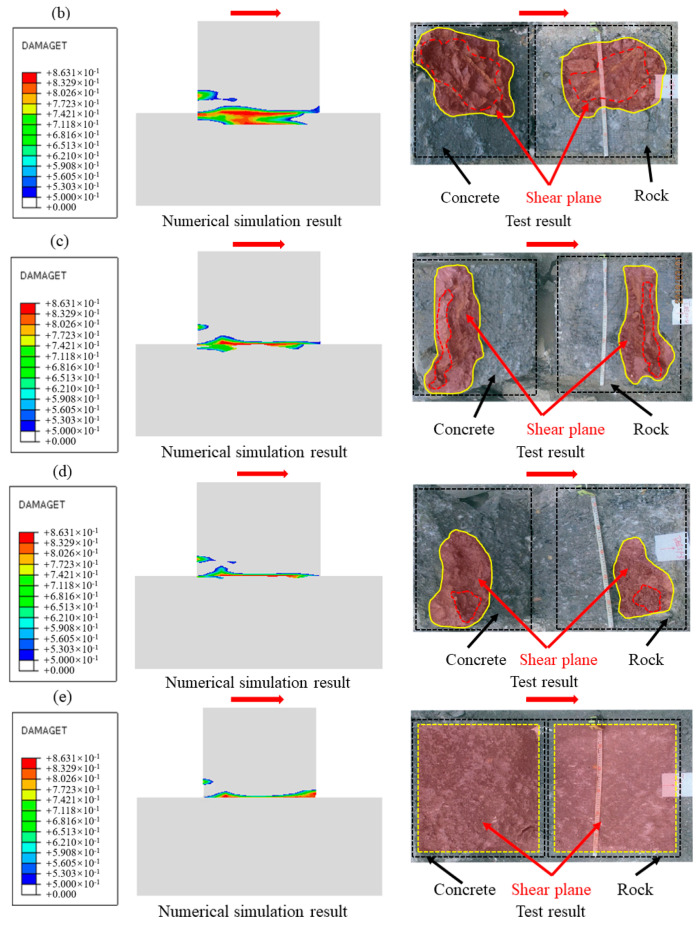
The failure mode of cementation surface: (**a**) normal stress 0.28 MPa, (**b**) normal stress 0.58 MPa, (**c**) normal stress 0.88 MPa, (**d**) normal stress 1.20 MPa, (**e**) normal stress 1.58 MPa.

Due to the influence of the rock mass’s normal stress, joint, and structural characteristics, the shear failure plane may not develop completely along the cementation surface. The observation of the shear failure surface of the test shows that the failure forms of the cementation surface under different normal stresses are different. Figure 25 shows the comparison of numerical simulation and cross-sectional view of the in-situ direct shear test, and the failure mode of the shear surface can be summarized into three categories. The first type is shear failure along the cementation surface; that is, the shear plane develops through the cementation surface, resulting in shear sliding; the shear plane is relatively flat, such as the specimen under the working condition of normal stress of 1.50 MPa. The mechanism of this failure form is that when the normal stress is large, the shear strength of the cementation surface is significantly smaller than the strength of the concrete and the rock mass itself. The damage is first generated at the loading end. Then, shear yield occurs at the cementation surface and then gradually expands along the shear direction, forming a penetration zone when the stress reaches the peak, and finally failure occurs under the action of shear stress. The second type is mixed shear failure, in which a part of the shear surface extends along the joint fractures of the rock mass and staggers with the cementation surface to form a “wave”-type failure surface, such as the specimen under normal stress of 0.88 MPa and 1.20 MPa. The mechanism of this failure form is that under the general normal stress, the shear strength of the cementation surface is similar to that of bedrock, and the small pieces of gravel or microcracks on the surface of bedrock make the shear failure surface expand along part of the rock mass, and under the action of shear stress, the underlying rock mass is brought out, and mixed failure occurs. The third type is shear failure along the bedrock, which is manifested as serious convex shear facing the bedrock; a larger volume of rock mass is brought out, and the morphology of the shear surface is different, such as the specimen under normal stress of 0.28 MPa and 0.58 MPa. The mechanism of this failure form is that under the action of low normal stress, the joints and initial fractures inside the rock make its shear strength lower than that of the cementation surface, and stress concentration often occurs at the tip of the crack inside the bedrock under load, and after reaching the critical value, the fracture will gradually expand under the action of shear stress to form a penetration zone, so that the fracture surface will fail along the inside of the bedrock. Through in-situ direct shear tests and numerical simulation, the proportion of the shear surface area is shown in Table 5 It can be found that the failure mode is related to the magnitude of normal stress. With the increase in normal stress, the shear plane along the cementation surface increases gradually, and the fracture surface tends to flatten, which is due to the increase in normal stress to increase the occlusion force of the internal joint surface of the bedrock. This phenomenon results in the fragmentation of cementitious materials and the consequent loss of cohesive strength. so that the failure surface mainly develops along the cementation surface. The asperities of rough joints exhibit a dilation-induced climbing effect during shearing, requiring additional work expenditure for slip initiation, which manifests as enhanced peak shear strength. With increasing shear displacement, stress redistribution occurs from bonded zones to intact regions. In the Concrete Damage Plasticity (CDP) model, this mechanism is explicitly represented through the evolution of damage variables that dynamically modulate material stiffness, thereby facilitating stress transfer from damaged to undamaged domains. The error analysis of the data shows that the numerical simulation results are consistent with the test results, and the error is less than 10%, which is within a reasonable range. The results can meet the requirements of numerical analysis, and the feasibility of the numerical simulation method is verified. The above research on the failure mode of cementation surface can provide a reference for relevant tests and engineering.

## 6. Conclusions

Through numerical simulation, the distribution law of mechanical properties and damage evolution process at the cementation surface of concrete-bedrock are analyzed, and the damage evolution law and failure mechanism are revealed, and the main conclusions are as follows.

(1) The shear stress-shear displacement relationship curve at the concrete-bedrock cementation surface demonstrates an initial ascending trend followed by a gradual softening phase. Numerical simulations yield peak shear strength and shear strength parameters with relative errors below 10%, while accurately replicating the experimental failure modes. These results validate the numerical approach as an effective methodology for investigating the failure mechanisms and damage evolution of concrete-bedrock surfaces, elucidating the complete damage progression from initiation, accumulation, and propagation to final rupture at the cementation plane.
(2) The failure modes under various loading conditions can be classified into three distinct categories: surface failure, composite shear failure, and bedrock failure. Surface failure exhibits a relatively planar shear surface with fractures propagating along the cementation plane. Composite shear failure manifests as partial shear propagation along rock mass discontinuities, intersecting with the surface to form an undulating failure surface. Bedrock failure features pronounced shear penetration into the intact rock matrix, resulting in substantial surface asperities.
(3) The experimental data obtained in this study are relatively limited. In subsequent research, we will conduct more extensive studies to acquire additional data for further refinement of the constitutive model. Meanwhile, a more sophisticated model will be established to enable deeper investigation.

## Figures and Tables

**Table 1:** Test site label of the in-situ direct shear test.

Dam Block	Test Site Label				
3^#^ dam block	3-1	3-2	3-3	3-4	3-5
4^#^ dam block	4-1	4-2	4-3	4-4	4-5
5^#^ dam block	5-11	5-12	5-13	5-14	5-15
5^#^ dam block	5-21	5-22	5-23	5-24	5-25
6^#^ dam block	6-1	6-2	6-3	6-4	6-5

**Table 2:** Material parameters of finite element model.

Material	Density	Elastic Modulus	Poisson’s Ratio	ψ	ε	$\sigma_{b0}$	$K_{c}$	μ
Concrete	2400 kg/m^3^	25,500 MPa	0.167	30	0.1	1.16	0.667	0
Bedrock	2710 kg/m^3^	12,900 MPa	0.23					

Note: In the table, ψ is the dilation angle; ε is the eccentricity; $\sigma_{b0}$ is the ratio of biaxial compressive strength to uniaxial compressive strength of concrete; $K_{c}$ is the influence parameter for the yield mode of concrete; and μ is the viscosity parameter.

**Table 3:** The setting of shear load analysis steps under different working conditions.

Normal Load MPa	Shear Load/MPa				
	Step-1	Step-2	Step-3	Step-4	Step-5
0.28	1.5	2	4	2	1
0.58	1.5	2	4	3	1.5
0.88	1.5	4	4	4	4
1.20	1.5	6	6	6	6
1.50	2.5	7	5	4	4

**Table 4:** Fitting parameters.

Normal Stress/MPa	Slope	Intercept	Correlation Coefficient
0.28	1.26	−0.36	0.93
0.58	1.14	−0.45	0.97
0.88	0.85	−0.46	0.98
1.20	0.89	−0.64	0.99
1.50	0.73	−0.47	0.90

**Table 5:** The proportion of the shear surface area.

Normal Stress MPa	Test Result		Simulation Result		Error
	Cementation Surface	Bedrock	Cementation Surface	Bedrock	
0.28	55%	45%	58%	42%	5.5%
0.58	70%	30%	65%	35%	−7.1%
0.88	85%	15%	80%	20%	−5.9%
1.20	80%	20%	80%	20%	0
1.50	90%	10%	97%	3%	7.8%

## Data Availability

The original contributions presented in this study are included in the article. Further inquiries can be directed to the corresponding author.
